# Impact of duty hours on competency‐related knowledge acquisition among community hospital residents

**DOI:** 10.1002/jgf2.594

**Published:** 2022-11-23

**Authors:** Kazuya Nagasaki, Yuji Nishizaki, Chisato Hachisuka, Tomohiro Shinozaki, Taro Shimizu, Yu Yamamoto, Kiyoshi Shikino, Sho Fukui, Sho Nishiguchi, Kohta Katayama, Masaru Kurihara, Hiroyuki Kobayashi, Yasuharu Tokuda

**Affiliations:** ^1^ Department of Internal Medicine, Mito Kyodo General Hospital University of Tsukuba Ibaraki Japan; ^2^ Division of Medical Education Juntendo University School of Medicine Tokyo Japan; ^3^ Department of Information and Computer Technology, Faculty of Engineering Tokyo University of Science Tokyo Japan; ^4^ Department of Diagnostic and Generalist Medicine Dokkyo Medical University Hospital Tochigi Japan; ^5^ Division of General Medicine, Center for Community Medicine Jichi Medical University Tochigi Japan; ^6^ Department of General Medicine Chiba University Hospital Chiba Japan; ^7^ Department of Emergency and General Medicine Kyorin University Chiba Japan; ^8^ Department of General Internal Medicine Shonan Kamakura General Hospital Kamakura Japan; ^9^ Division of General Internal Medicine, Department of Internal Medicine St. Marianna University School of Medicine Kawasaki Japan; ^10^ Department of Patient Safety Nagoya University Hospital Nagoya Japan; ^11^ Muribushi Okinawa for Teaching Hospitals Okinawa Japan; ^12^ Tokyo Foundation for Policy Research Tokyo Japan

**Keywords:** clinical competency, community hospital, duty hour restriction, General Medicine In‐training Examination, Japan, postgraduate resident

## Abstract

**Background:**

The effect of duty hour (DH) restrictions on postgraduate residents' acquisition of clinical competencies is unclear. We evaluated the relationship between DHs and competency‐related knowledge acquisition using the General Medicine In‐training Examination (GM‐ITE).

**Methods:**

We conducted a multicenter, cross‐sectional study of community hospital residents among 2019 GM‐ITE examinees. Self‐reported average DHs per week were classified into five DH categories and the competency domains were classified into four areas: symptomatology and clinical reasoning (CR), physical examination and clinical procedure (PP), medical interview and professionalism (MP), and disease knowledge (DK). The association between these scores and DHs was examined using random‐intercept linear models with and without adjustment for confounding factors.

**Results:**

We included 4753 participants in the analyses. Of these, 31% were women, and 49.1% were in the postgraduate year (PGY) 2. Mean CR and MP scores were lower among residents in Category 1 (<50 h) than in residents in Category 3 (≥60 and <70 h; reference group). Mean DK scores were lower among residents in Categories 1 and 2 (≥50 and <60 h) than in the reference group. PGY‐2 residents in Categories 1 and 2 had lower CR scores than those in Category 3; however, PGY‐1 residents in Category 5 showed higher scores.

**Conclusions:**

The relationship between DHs and each competency area is not strictly linear. The acquisition of knowledge of physical examination and clinical procedures skills in particular may not be related to DHs.

## Graphical abstract

In this study, we examined the relationship between duty hour (DH) and scores in each competency area of the General Medicine In‐training Examination among residents affiliated with community hospitals in Japan. We found that acquiring knowledge of clinical reasoning medical interview skills professionalism and disease‐related topics requires a certain level of DHs whereas that related to physical examination and clinical procedures may not be related to DHs.

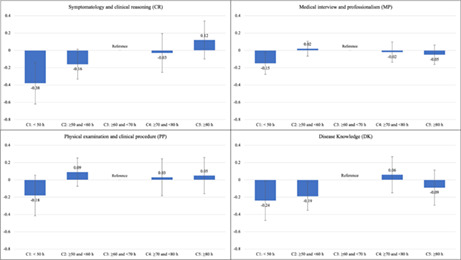

## 1 INTRODUCTION

Since 2018, workstyle reforms in Japan have affected all workers, including physicians and postgraduate residents.^1^ Among them, reducing long working hours is considered to be one of the most important measures to improve the working environment. The Ministry of Health, Labor, and Welfare (MHLW) decreed that the upper limit of duty hours (DH) for physicians should be 960 h per year (equivalent to a 60‐h work week), starting in 2024, while the limit for residents was set at 1860 h per year (equivalent to an 80‐h work week), from an educational perspective. However, this decision remains questionable because of concerns about residents' health, as well as the lack of sufficient evidence on the relationship between DHs and educational outcomes.

There has been minimal research on the association between the number of DHs and resident competencies in Japan as well as globally. One report analyzed the association between resident DHs and in‐training examination scores in Japan.^2^ The study demonstrated that fewer than 60–65 DHs per week were independently associated with lower performance; however, exceeding 65 DHs per week did not improve performance.^2^ Another research further reported that Japanese residents with fewer than 60 DHs per week tended to spend less time in self‐study than did those with more than 65 DHs per week.^3^ Surveys about the impact of DH restrictions on residents in the United States found that, while resident health and burnout improved, there were negative effects on resident education and patient care.^4^, ^5^, ^6^ These studies assessed the overall impact on resident education and suggested that DH restrictions had negative impacts on education. A systematic review researching the effect of DH restrictions on postgraduate medical education found that the effect on education was assessed mostly by measuring access to educational opportunities, total in‐training examination scores, and caseloads.^7^ In this context, we found no previous studies that directly examined the impact on DHs and residents' competency in various areas. Investigating the relationship between DHs and each resident's competency could prompt program directors, training program developers, and policymakers to consider ways to mitigate the negative educational effects of DH restrictions.

In this study, we examined the relationship between DHs and scores in each competency area of the General Medicine In‐training Examination (GM‐ITE), an in‐training examination in Japan among residents affiliated with community hospitals in Japan.

## 2 METHODS

### 2.1 Study populations

In Japan, there is a 2‐year postgraduate clinical training system. We conducted a multicenter, cross‐sectional study of Japanese residents in postgraduate year 1 (PGY‐1) and postgraduate year 2 (PGY‐2) of their training. Before conducting the survey, we obtained written informed consent for study participation from all participants. The research consent document stated that the questionnaire results would be anonymized. An appropriate research ethics committee approved the study. This study followed the Strengthening the Reporting of Observational Studies in Epidemiology (STROBE) guidelines.

In Japan, teaching hospitals are divided into three categories: *independent* hospitals, *administrative* hospitals, and *cooperative* hospitals.^8^, ^9^ *Independent* hospitals provide training programs almost exclusively on their own, while *administrative* hospitals operate programs in collaboration with *cooperative* hospitals. Many university hospitals are *administrative* hospitals, and many of them have programs, so‐called tasukigake programs, where training at one *cooperative* hospital lasts 1 year. Thus, the work styles of university hospital residents are more diverse than those of community hospital residents. Because this study used the average DHs of the entire training program for analysis, university hospital residents, whose DHs would be difficult to interpret, were excluded from the study, and only community hospital residents were included in our study.

As part of a training environment questionnaire completed immediately after the examination, participants were asked about average DHs during their residency program. The questionnaire also included the number of emergency department (ED) duties per month, the mean number of assigned inpatients, and self‐study time per day.

### 2.2 Measurements

The primary independent variable in this study was the average self‐reported DHs per week. We defined DHs as the total hours of weekday work duty, night ED duty (including restrained time when not providing medical care), and weekend work duty. DHs were answered in eight categories, which were reorganized into five categories with 10‐h increments: Category 1 (<50 h), Category 2 (≥50 and <60 h), Category 3 (≥60 and <70 h), Category 4 (≥70 and <80 h), Category 5 (≥80 h). Category 5 exceeded the maximum DHs for residents set by the MHLW.^1^

Knowledge acquisition of resident competencies was assessed using the scores of the GM‐ITE. The GM‐ITE is an in‐training examination conducted in Japan. It is administered each year by the Japan Institute for Advancement of Medical Education Program (JAMEP, a nonprofit organization in Tokyo, Japan), and training programs voluntarily participate in the examination. The GM‐ITE is designed to provide objective and reliable clinical knowledge assessment of postgraduate residents, with results shared with the residents and their residency programs.^10^, ^11^

The examination contains 60 multiple‐choice questions, which are grouped into four major competency areas: symptomatology and clinical reasoning (CR, 18 questions), physical examination and clinical procedure (PP, 18 questions), medical interview and professionalism (MP, 6 questions), and disease knowledge (DK, 18 questions). Although both CR and DK areas contain case‐based questions on diagnosis and management, CR mainly focuses on the diagnosis, and DK focuses on management. In the PP area, we applied audio and video questions. We excluded one question in the PP area after the examination as an inappropriate question. Participants completed the GM‐ITE from January 21 to 28, 2020.

### 2.3 Statistical analyses

We examined the association between resident DHs and each competency area score (one point per question) using random‐intercept linear models, accounting for hospital variability as normal random intercepts. Category 3 was set as the DH reference for the analyses, considering that 60 h per week was the basic upper limit of DHs for all doctors.^1^ We excluded residents who did not provide information about DHs from the analyses. We repeated the analyses, stratified by PGY. We adjusted for the following variables that could potentially be associated with the total GM‐ITE score: sex, PGY, ED duties, number of assigned inpatients, and self‐study time.^2^, ^12^ All analyses were performed using SAS version 9.4 (SAS Inst.).

## 3 RESULTS

The GM‐ITE survey included 6869 postgraduate residents from 539 residency programs. The survey response rate was 89.7% (6164/6869). We excluded 571 residents who did not provide DH information, and also excluded another 840 university and university‐affiliated hospital residents. Finally, the analysis included 4753 participants. Among them, 31% were women and 50.9% were PGY‐1. The mean (± standard deviation) of total GM‐ITE scores of all the participants was 29.7 ± 5.3. The scores in the various competency areas were as follows: CR, 9.9 ± 2.3; PP, 9.2 ± 2.3; MP, 2.9 ± 1.2; and DK, 8.2 ± 2.2. Table 1 summarizes resident characteristics categorized by DHs, and Table S1 in Appendix S1 lists them stratified by PGY.

**TABLE 1:** Characteristics. Baseline characteristics of residents categorized by DHs

Variable	C1: <50 h	C2: ≥50 and <60 h	C3: ≥60 and <70 h	C4: ≥70 and <80 h	C5: ≥80 h
	*N* = 502	*N* = 1393	*N* = 1542	*N* = 580	*N* = 736
Male sex (%)	65.1	69.6	69.5	66.7	70.9
PGY‐2 (%)	52.2	47.2	48.6	49.8	51.4
ED duties per month (%)					
None	6.2	1.5	1	1.7	0.8
1–2	24.8	13.7	7.6	7.2	4.6
3–5	62.7	78.3	79.2	74.3	66.3
6 or more	6	6.1	11.6	16.6	27.6
Unknown	0.4	0.4	0.6	0.2	0.7
Assigned inpatients (%)					
0–4	30.3	21.4	16	13.3	9.9
5–9	60.4	65.4	64.4	60.7	55
10–14	6.6	9	13.7	17.8	18.4
15 or more	1.4	1.2	2.5	5.2	14
Unknown	1.4	3.1	3.3	3.1	2.7
Self‐study time per day (%)					
None	3.4	3.4	3.4	4	6.2
0–30 min	49.5	37.1	34.3	30.3	32.9
31–60 min	36.5	43.6	44.3	43.8	38.5
61–90 min	9.2	13.1	14.9	17.8	17.5
91 min or more	1.4	2.7	3.1	4.1	4.9

*Note*: Calculation of the average duty hours was based on the sum of weekday work duty, night emergency department (ED) duties, and weekend work duty.

Abbreviations: C1–C5, Category 1 to Category 5; DHs, duty hours; ED, emergency department; PGY, postgraduate year.

**FIGURE 1:**
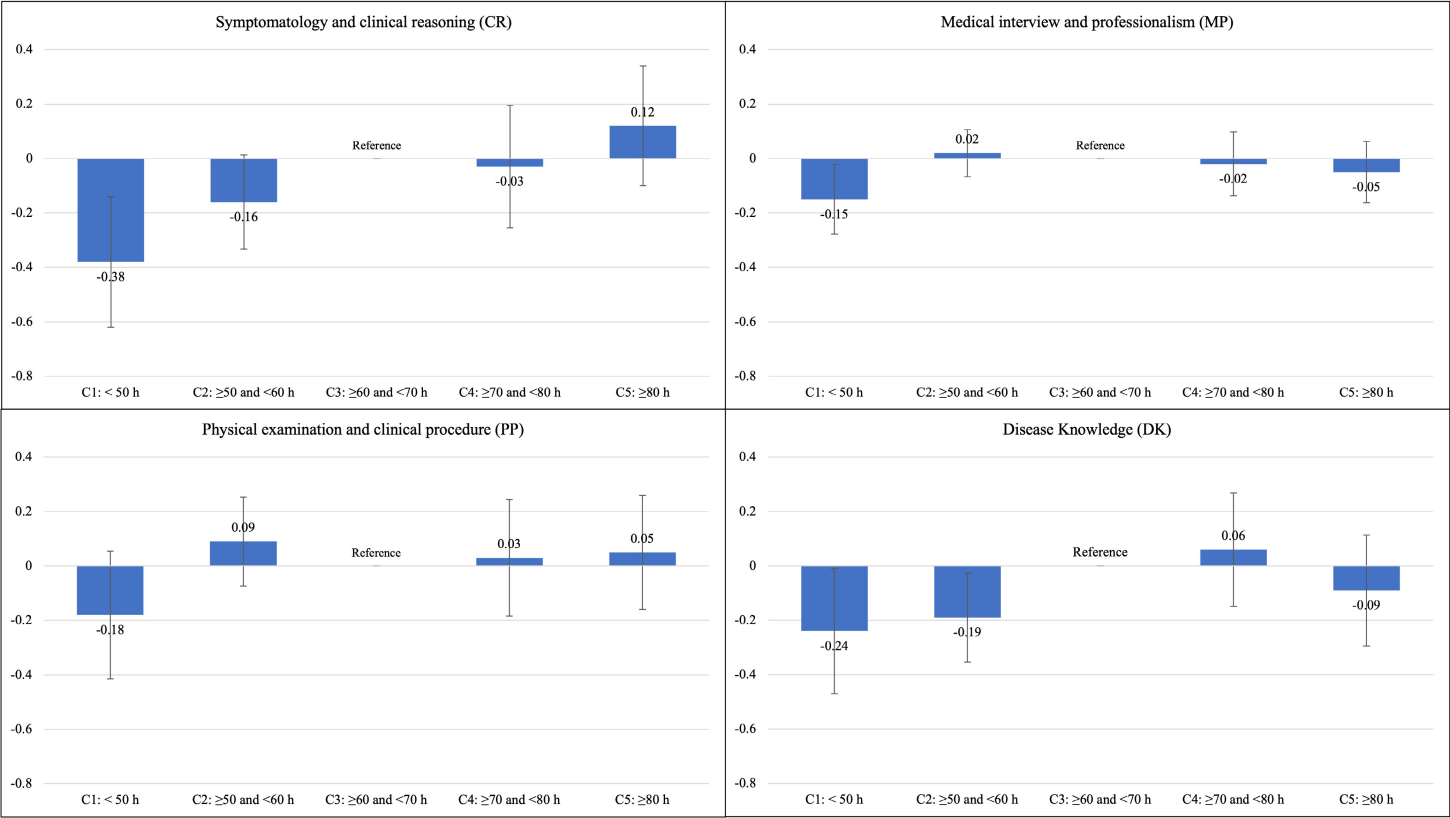
Estimates of mean score differences for each subcategory score between residents' duty hour categories, with multivariable adjustments. *Note*: Non‐responders for Emergency Department duties and inpatients were included in “unknown.” Non‐responders for self‐study time were excluded from the multivariable analysis. Error bars indicate 95% confidence interval. C1–C5, Category 1 to Category 5.

Figure 1 and Table S2 in Appendix S2 present the results of the analyses assessing the association between resident DHs and each competency area score. Compared with residents in Category 3, mean CR scores were lower among residents in Category 1 (mean score difference [MSD], −0.46; 95% CI, −0.70 to −0.23; *p* < 0.001) and Category 2 (−0.19; −0.36 to −0.03; *p* = 0.02). Similarly, mean DK scores were lower among residents in Category 1 (−0.33; −0.55 to −0.11; *p* = 0.004) and Category 2 (−0.22; −0.37 to −0.06; *p* = 0.01) than in residents in Category 3. Compared with the Category 3 group, Category 1 residents scored lower on PP (−0.26; −0.49 to −0.03; *p* = 0.03) and MP (−0.15; −0.28 to −0.03; *p* = 0.01).

After adjusting for possible confounding variables, mean DK scores were lower among residents in Category 1 (−0.24; −0.47 to −0.02; *p* = 0.03) and Category 2 (−0.19; −0.35 to −0.03; *p* = 0.02) than among residents in Category 3. Compared with the Category 3 group, Category 1 residents scored lower on CR (−0.38; −0.62 to −0.15; *p* = 0.02) and MP (−0.15; −0.27 to −0.02; *p* = 0.02). After adjustment, there was little or no mean score difference among the DH Categories in the PP competency areas.

**FIGURE 2:**
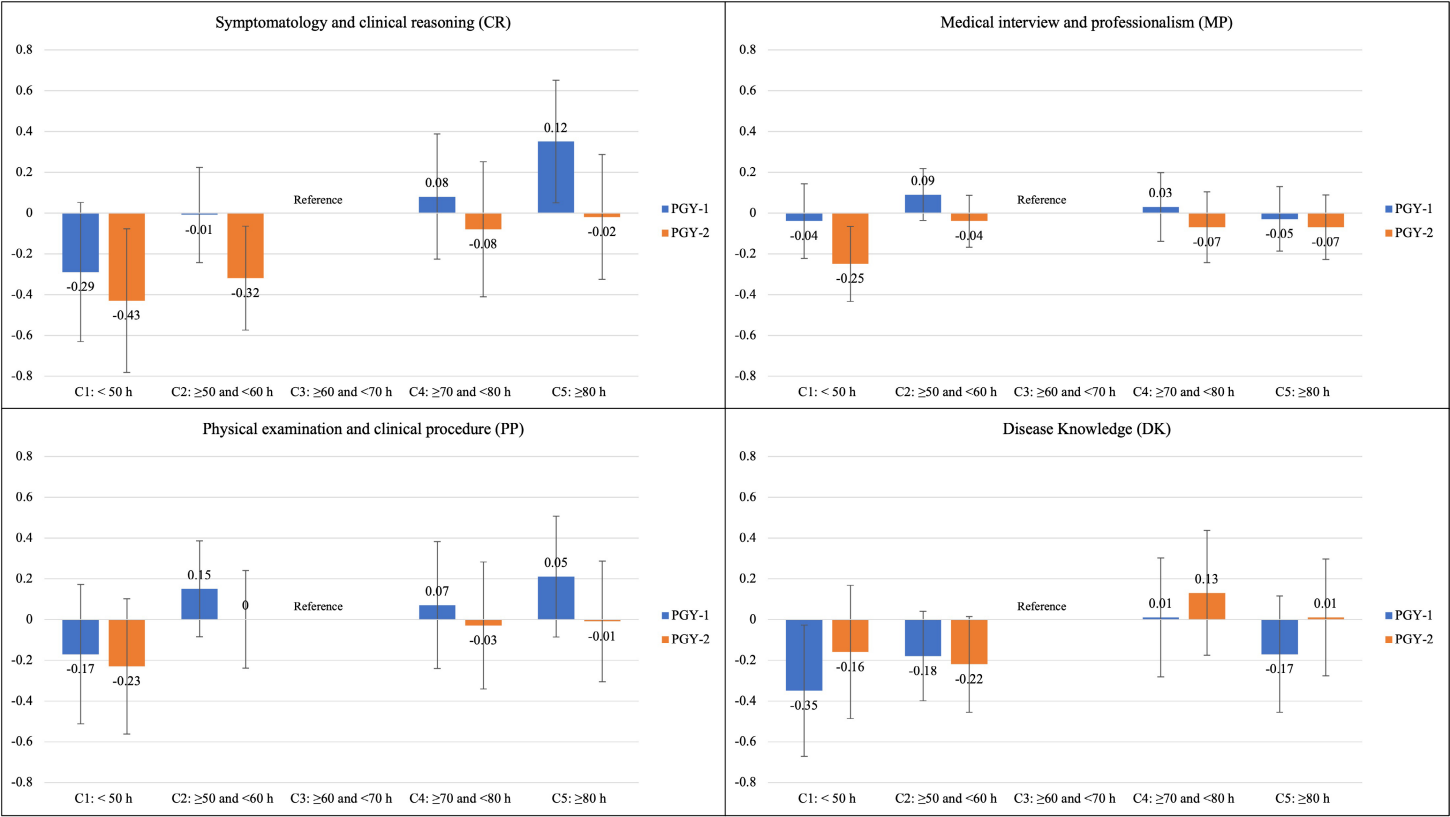
Estimates of mean score differences for each subcategory score between categories of residents' duty hours, with multivariable adjustments stratified by postgraduate year. *Note*: Non‐responders for Emergency Department duties and inpatients were included in “unknown.” Non‐responders for self‐study time were excluded from the multivariable analysis. Error bars indicate 95% confidence interval. C1–C5, Category 1 to Category 5.

Figure 2 and Table S3 in Appendix S2 present the adjusted results of the association between resident DHs and each competency area score stratified by PGY. PGY‐2 residents in Categories 1 and 2 had lower CR scores than those in Category 3; however, PGY‐1 residents in Category 5 had higher CR scores than those in Category 3. DK scores were lower for PGY‐1 Category 1 residents than for the reference category residents, but there was no difference in DK scores between categories for PGY‐2 residents. MP scores were lower for PGY‐2, but not for PGY‐1, Category 1 residents than for the reference category residents. There was no difference in mean scores between each DH Category for PGY‐2 and PGY‐1 residents in the PP competency areas.

## 4 DISCUSSION

In this study of the effect of DHs on residents' competencies in different areas, we found that, overall, community hospital residents with <60 DHs per week had lower DK scores on the GM‐ITE than those with 60–70 DHs per week, whereas those with >70 DHs per week did not have higher scores. Furthermore, <50 DHs were associated with lower CR and MP scores. When we stratified the analysis by PGY, CR scores decreased for residents in the ≤60 DHs group for PGY‐2 and increased for those in the ≥80 DHs for PGY‐1. In residents with <50 DHs, the DK scores decreased only for PGY‐1 residents, and the MP scores decreased only for PGY‐2 residents. Thus, there were discrepancies in the association between DHs and knowledge acquisition and its application in each competency area.

Knowledge acquisition for the competency areas of DK and CR was found to be related to resident DHs, with a positive trend up to 60–70 DHs per week, but no increase in the score when DHs exceeded 70 h. For CR knowledge acquisition, working more during PGY‐1 temporarily raised the score, but for those in PGY‐2, the scores of residents who worked more than 60 h per week eventually seemed to reach a plateau. Nevertheless, reducing DHs of PGY‐1 could be a barrier to CR skill acquisition. Although these adjusted score differences are about 2%–4% in each category, the “cumulative” difference in scores by DHs was not negligible, as the resident DH policies affect the working conditions of almost all residents. These two competency areas can be assessed by written examinations, and are generally the primary assessment items on the in‐training examination. There was no clear upward or downward trend in the impact of work‐hour restrictions on the resident performance in in‐training examinations in the United States in the fields of internal medicine, emergency medicine, and surgery.^13^, ^14^, ^15^, ^16^ The current variation in DHs in Japan may reflect the number of assigned inpatient cases. The number of patient encounters correlated with performance in in‐training examinations in internal medicine residents, family medicine residents, and students on internal medicine clerkships.^17^, ^18^, ^19^ Future research should examine the relationship between resident DHs and the number of clinical encounters, and the relationship between these and residents' clinical knowledge in Japan.

The relationship between knowledge acquisition and work hours in the MP and PP competency areas showed a different relationship than that in the CR and DK areas. MP scores appeared to be low for residents who worked less than 50 h per week, particularly in the PGY‐2. A 50‐h work week means almost no overtime work, which implies that weekend work and nighttime ED duties are almost nonexistent. This is quite different from the work style of a typical hospital doctor, and such a learning environment is unlikely to foster professionalism. On the contrary, DHs appeared to have no effect on the PP scores. Clinical training in Japan may not provide sufficient education on these competency areas. In particular, previous studies have pointed out a lack of education on professionalism.^20^ Another possible explanation is that DH variation may have both positive and negative effects on these competencies, and the effects may be offset.^21^

For scores in the PP area, there was no clear relationship between DHs and the acquisition of knowledge. There are two possible explanations for this observation. First, Japanese clinical residents may have acquired sufficient knowledge of these competency areas during medical school training. Medical students in Japan must pass an Objective Structured Clinical Examination, which evaluates skills in physical examination, medical interviewing, and clinical procedures. Second, postgraduate training may not adequately teach these competencies, particularly physical examination skills.^22^ Even if there is a large volume of case experience, teaching and feedback may not be sufficient, possibly resulting in a lack of improved performance of residents.

In this study, we found that working more than 60–70 h per week did not increase scores in any competency area for PGY‐2 residents nearing the end of their training, although the relationship between scores in each of the competency areas and DHs was different. McCoy et al. proposed four models for the relationship between patient encounter volume and knowledge acquisition: a linear relationship, threshold, Yerkes–Dodson curve (positive relationship up to a certain point, negative relationship thereafter), and null.^19^ In their study, the patient encounter volume and in‐training examination scores were positively associated, but in our study, we found that the relationship of DHs with CR and DK scores had a threshold. The in‐training examination score of residents may improve as the patient encounter volume increases but increasing DHs further will not improve scores. Therefore, it may be necessary to set an appropriate upper limit for DHs. Our recent study also found that self‐study time was shorter in residents who worked less than 60–65 h per week.^3^ Taken together with the findings of this study, 60–70 h per week is a benchmark for an appropriate upper limit on DHs. However, residents' health and patients' safety are also important factors in determining DHs. Thus, further research will be needed in the future.

There were several limitations in this study. First, the number of questions for each competency area is small and may not provide a reliable assessment. In general, in‐training examinations are reportedly adequate, with a reliability coefficient of 0.80 at around 100 questions; however, the GM‐ITE may not be as accurate, because it contains 60 questions in total and fewer questions for each competency area.^23^ Additionally, there are some concerns about the validity of the GM‐ITE itself. A previous study showed that GM‐ITE scores correlated well with those of a foreign examination, but their relationship to actual clinical performance has not been investigated.^11^ Second, the GM‐ITE is a written test and, by its nature, can only assess knowledge and related cognitive abilities. Therefore, GM‐ITE scores are not necessarily a guarantee of actual clinical competence. In this study, the areas of physical examination, clinical skills, communication, and professionalism were assessed by evaluating the acquisition of knowledge about them and their application. However, we do not know whether this reflects performance in real clinical practice. Third, DHs were self‐reported in the survey and may not have been reported accurately. The trend of bias in self‐reported DHs may be different for each group. In addition, residents who did not report their working hours (571 excluded individuals) performed worse on the examination than those who reported them (mean 29.7 [SD 5.3] vs. 28.9 [SD 5.6]). The residents who performed worse in the examination were more likely to refuse to participate in the study. Fourth, on average, only one‐third of all residents take the GM‐ITE, which may introduce selection bias. Participation in GM‐ITE is voluntary in training hospitals, which may be more education‐oriented, and residents in‐training hospitals may be more motivated to improve their skills. In addition, we excluded university hospital residents from our analysis as they tend to have greatly variable working patterns. To allow for a comparison of residents at university and community hospitals, the baseline characteristics of the university hospital residents are shown in Table S4 in Appendix S3. A summary of the examination scores is presented in Table S5 in Appendix S4. University hospital residents appear to have less ED duty, fewer assigned inpatients, less time for self‐study, and lower scores than community hospital residents. Fifth, the study did not assess residents' baseline clinical knowledge. Therefore, to measure the impact of working hours on resident improvement directly, adjusting for baseline GM‐ITE scores, national examination scores, and performance during medical school is necessary. Sixth, some community hospitals also have “tasukigake” programs. Thus, there are residents in community hospitals who work in a variety of work locations similar to those in university hospitals. However, this study did not examine the number of community hospital residents who partook in “tasukigake” programs.

In conclusion, our results suggest that the knowledge acquisition of CR, medical interviews, professionalism, and disease‐related topics requires a certain level of DHs, while the application of knowledge of physical examination and procedures may not be related to DHs. In addition, the limit of 80 DHs per week starting in 2024 will have only a limited impact on the residents' acquisition of competency‐related knowledge. In the future, these competencies should be evaluated using more suitable assessment methods, and their relationships with DHs should be investigated in more detail.

## CONFLICT OF INTEREST

The JAMEP was involved in collecting and managing data as the GM‐ITE administrative organization. It did not participate in designing and conducting the study; data analysis and interpretation; preparation, review, or approval of the manuscript; and the decision to submit the manuscript for publication. Dr. Nishizaki received an honorarium from the JAMEP as the GM‐ITE project manager. Dr. Tokuda is one the JAMEP directors. Dr. Kobayashi received an honorarium from the JAMEP as a speaker for the JAMEP lecture. Dr. Shimizu and Dr. Yamamoto received an honorarium from the JAMEP as exam preparers of the GM‐ITE. Dr. Nishizaki, Dr. Tokuda, Dr. Kobayashi, Dr. Shimizu, and Dr. Yamamoto were not involved in the analysis.

## ETHICS APPROVAL STATEMENT

The Ethics Review Board of Juntendo University School of Medicine approved the study.

## PATIENT CONSENT STATEMENT

We obtained informed consent to participate in this study from all participants before the survey. The research consent document stated that the questionnaire results would be anonymized.

## Supporting information


Appendix S1–S4

## Data Availability

Due to the nature of this research, participants of this study did not agree for their data to be shared publicly, so supporting data are not available.
