# Supplementary material for: Impact of duty hours on competency‐related knowledge acquisition among community hospital residents
Source: J Gen Fam Med. 2022 Nov 23;24(2):87–93. doi: 10.1002/jgf2.594 (PMC10000279; doi:10.1002/jgf2.594)
Supplement: Supplementary file 1 — Appendix S1–S4 [file JGF2-24-87-s001.docx]

**Online Supplemental Materials**

**Supplemental material for “Impact of Resident Duty Hours on Competency-Related Knowledge Acquisition”**

**This supplemental material comprises the following sections:**

**Appendix S1.** Baseline characteristics of participants categorized by postgraduate year

**Appendix S2.** Estimates of mean score differences for each subcategory score between categories of residents’ duty hours

**Appendix S3**. Baseline characteristics of the university hospital residents

**Appendix S4**. Summary of examination scores

**Appendix S1**

**Supplemental Table 1.** Baseline characteristics of participants categorized by postgraduate year

| Variable |  | PGY-1 | PGY-2 |
| --- | --- | --- | --- |
|  |  | N=2417 | N=2336 |
| Male sex (%) |  | 70.5 | 67.3 |
| ED duties per month (%) | |  |  |
| None |  | 1.9 | 1.7 |
| 1–2 |  | 11.8 | 9.5 |
| 3–5 |  | 74.5 | 74.7 |
| 6 or more |  | 11.3 | 13.7 |
| Unknown |  | 0.5 | 0.4 |
| Assigned inpatients (%) | |  |  |
| 0–4 |  | 19.1 | 16.5 |
| 5–9 |  | 61.9 | 62.9 |
| 10–14 |  | 12.6 | 13.0 |
| 15 or more |  | 3.6 | 4.7 |
| Unknown |  | 2.9 | 3.0 |
| Self-study time per day (%) | |  |  |
| None |  | 3.8 | 4.1 |
| 0–30 min |  | 39.1 | 32.8 |
| 31–60 min |  | 40.5 | 44.1 |
| 61–90 min |  | 13.1 | 16.1 |
| 91 min or more | | 3.4 | 3.0 |

Note: Abbreviations: PGY = postgraduate year; ED = emergency department.

**Appendix S2.** Estimates of mean score differences for each subcategory score between categories of residents’ duty hours

In this section, we showed the estimates of mean score differences for each subcategory score between categories of residents’ duty hours (Supplementary Table 2). We categorized these score differences according to postgraduate years (Supplementary Table 3). In addition, we demonstrated the estimates of mean score differences for each subcategory score between original 8 categories of residents’ duty hours (Supplementary Table 4).

**Supplemental Table 2**. Estimates of mean score differences for each subcategory score between categories of residents’ duty hours

|  |  | Duty hour Category | | | | |
| --- | --- | --- | --- | --- | --- | --- |
| The examination subcategory | | C1: < 50 h | C2: ≥50 and <60 h | C3: ≥60 and <70 h | C4: ≥70 and <80 h | C5: ≥80 h |
| *N* |  | 502 | 1393 | 1542 | 580 | 736 |
| *Symptomatology and clinical reasoning (CR)* | | | |  |  |  |
|  | Mean±SD | 9.42±2.32 | 9.72±2.33 | 9.93±2.32 | 9.98±2.31 | 10.17±2.38 |
|  | Difference (95% CI) | -0.46 (-0.70, -0.23) | -0.19 (-0.36, -0.03) | Reference | -0.01 (-0.23, 0.21) | 0.15 (-0.06, 0.36) |
|  | P | 0.00 | 0.02 |  | 0.95 | 0.15 |
|  | Adjusted difference (95% CI) | -0.38 (-0.62, -0.15) | -0.16 (-0.33, 0.01) | Reference | -0.03 (-0.25, 0.19) | 0.12 (-0.09, 0.34) |
|  | P | 0.00 | 0.06 |  | 0.80 | 0.25 |
| *Physical examination and clinical procedure (PP)* | | | |  |  |  |
|  | Mean±SD | 8.41±2.20 | 8.78±2.27 | 8.74±2.24 | 8.83±2.21 | 8.93±2.36 |
|  | Difference (95% CI) | -0.26 (-0.49, -0.03) | 0.06 (-0.10, 0.22) | Reference | 0.06 (-0.15, 0.28) | 0.07 (-0.13, 0.27) |
|  | P | 0.03 | 0.46 |  | 0.55 | 0.49 |
|  | Adjusted difference (95% CI) | -0.18 (-0.41, 0.05) | 0.09 (-0.07, 0.25) | Reference | 0.03 (-0.18, 0.24) | 0.05 (-0.15, 0.26) |
|  | P | 0.13 | 0.28 |  | 0.77 | 0.63 |
| *Medical interview and professionalism (MP)* | | | |  |  |  |
|  | Mean±SD | 2.81±1.29 | 2.99±1.15 | 2.96±1.23 | 2.94±1.17 | 2.91±1.24 |
|  | Difference (95% CI) | -0.15 (-0.28, -0.03) | 0.02 (-0.07, 0.11) | Reference | -0.03 (-0.14, 0.09) | -0.06 (-0.16, 0.05) |
|  | P | 0.01 | 0.62 |  | 0.66 | 0.31 |
|  | Adjusted difference (95% CI) | -0.15 (-0.27, -0.02) | 0.02 (-0.06, 0.11) | Reference | -0.02 (-0.14, 0.09) | -0.05 (-0.16, 0.06) |
|  | P | 0.02 | 0.60 |  | 0.68 | 0.37 |
| *Disease Knowledge (DK)* | | |  |  |  |  |
|  | Mean±SD | 7.90±2.24 | 8.04±2.14 | 8.27±2.15 | 8.38±2.13 | 8.24±2.30 |
|  | Difference (95% CI) | -0.33 (-0.55, -0.11) | -0.22 (-0.37, -0.06) | Reference | 0.09 (-0.12, 0.29) | -0.06 (-0.26, 0.13) |
|  | P | 0.00 | 0.01 |  | 0.42 | 0.51 |
|  | Adjusted difference (95% CI) | -0.24 (-0.47, -0.02) | -0.19 (-0.35, -0.03) | Reference | 0.06 (-0.15, 0.26) | -0.09 (-0.29, 0.11) |
|  | P | 0.03 | 0.02 |  | 0.59 | 0.36 |

Note: Adjusted for gender, postgraduate years, monthly Emergency Department (ED) duties, number of assigned inpatients, and self-study time. Non-responders for Emergency Department duties and inpatients were included in “unknown.” Non-responders for self-study time were excluded from the multivariable analysis. Abbreviations: C1–C5 = Category 1 to Category 5; SD = standard deviation; CI = confidence interval.

**Supplemental Table 3**. Estimates of mean score differences for each subcategory score between categories of residents’ duty hours stratified by postgraduate year

Postgraduate Year (PGY) 1

|  |  | Duty hour Category | | | | |
| --- | --- | --- | --- | --- | --- | --- |
| The examination subcategory | | C1: < 50 h | C2: ≥50 and <60 h | C3: ≥60 and <70 h | C4: ≥70 and <80 h | C5: ≥80 h |
| *N* |  | 240 | 735 | 793 | 291 | 358 |
| *Symptomatology and clinical reasoning (CR)* | | | |  |  |  |
|  | Mean±SD | 9.25±2.14 | 9.59±2.30 | 9.63±2.22 | 9.76±2.33 | 9.98±2.30 |
|  | Difference (95% CI) | -0.35 (-0.68, -0.02) | -0.02 (-0.25, 0.20) | Reference | 0.10 (-0.20, 0.40) | 0.34 (0.05, 0.63) |
|  | P | 0.04 | 0.86 |  | 0.50 | 0.02 |
|  | Adjusted difference (95% CI) | -0.29 (-0.63, 0.04) | -0.01 (-0.24, 0.22) | Reference | 0.08 (-0.22, 0.38) | 0.35 (0.06, 0.65) |
|  | P | 0.09 | 0.94 |  | 0.60 | 0.02 |
| *Physical examination and clinical procedure (PP)* | | | |  |  |  |
|  | Mean±SD | 8.35±2.18 | 8.77±2.27 | 8.64±2.22 | 8.79±2.24 | 8.90±2.38 |
|  | Difference (95% CI) | -0.24 (-0.56, 0.09) | 0.13 (-0.10, 0.35) | Reference | 0.11 (-0.19, 0.41) | 0.20 (-0.09, 0.48) |
|  | P | 0.15 | 0.27 |  | 0.48 | 0.18 |
|  | Adjusted difference (95% CI) | -0.17 (-0.50, 0.17) | 0.15 (-0.08, 0.38) | Reference | 0.07 (-0.23, 0.38) | 0.21 (-0.08, 0.50) |
|  | P | 0.33 | 0.20 |  | 0.63 | 0.16 |
| *Medical interview and professionalism (MP)* | | | |  |  |  |
|  | Mean±SD | 2.90±1.29 | 3.03±1.13 | 2.93±1.21 | 2.95±1.23 | 2.88±1.25 |
|  | Difference (95% CI) | -0.03 (-0.20, 0.14) | 0.09 (-0.03, 0.22) | Reference | 0.02 (-0.14, 0.18) | -0.05 (-0.20, 0.10) |
|  | P | 0.74 | 0.12 |  | 0.81 | 0.51 |
|  | Adjusted difference (95% CI) | -0.04 (-0.22, 0.14) | 0.09 (-0.04, 0.21) | Reference | 0.03 (-0.14, 0.19) | -0.03 (-0.18, 0.13) |
|  | P | 0.68 | 0.17 |  | 0.75 | 0.73 |
| *Disease Knowledge (DK)* | | |  |  |  |  |
|  | Mean±SD | 7.71±2.10 | 7.97±2.08 | 8.19±2.11 | 8.23±2.28 | 8.01±2.21 |
|  | Difference (95% CI) | -0.43 (-0.74, -0.13) | -0.19 (-0.41, 0.02) | Reference | 0.02 (-0.26, 0.31) | -0.20 (-0.47, 0.07) |
|  | P | 0.01 | 0.08 |  | 0.89 | 0.15 |
|  | Adjusted difference (95% CI) | -0.35 (-0.67, -0.04) | -0.18 (-0.39, 0.04) | Reference | 0.01 (-0.28, 0.29) | -0.17 (-0.45, 0.11) |
|  | P | 0.03 | 0.10 |  | 0.97 | 0.22 |

Postgraduate Year (PGY) 2

|  |  | Duty hour Category | | | | |
| --- | --- | --- | --- | --- | --- | --- |
| The examination subcategory | | C1: < 50 h | C2: ≥50 and <60 h | C3: ≥60 and <70 h | C4: ≥70 and <80 h | C5: ≥80 h |
| *N* |  | 262 | 658 | 749 | 289 | 378 |
| *Symptomatology and clinical reasoning (CR)* | | | |  |  |  |
|  | Mean±SD | 9.57±2.47 | 9.86±2.35 | 10.25±2.38 | 10.20±2.27 | 10.34±2.44 |
|  | Difference (95% CI) | -0.61 (-0.94, -0.27) | -0.38 (-0.62, -0.13) | Reference | -0.07 (-0.39, 0.25) | 0.03 (-0.27, 0.33) |
|  | P | 0.00 | 0.00 |  | 0.68 | 0.85 |
|  | Adjusted difference (95% CI) | -0.43 (-0.78, -0.09) | -0.32 (-0.57, -0.07) | Reference | -0.08 (-0.41, 0.24) | -0.02 (-0.32, 0.28) |
|  | P | 0.01 | 0.01 |  | 0.61 | 0.90 |
| *Physical examination and clinical procedure (PP)* | | | |  |  |  |
|  | Mean±SD | 8.46±2.22 | 8.79±2.28 | 8.85±2.27 | 8.87±2.18 | 8.97±2.35 |
|  | Difference (95% CI) | -0.31 (-0.63, 0.00) | -0.03 (-0.26, 0.21) | Reference | 0.02 (-0.28, 0.33) | 0.03 (-0.25, 0.32) |
|  | P | 0.05 | 0.82 |  | 0.88 | 0.82 |
|  | Adjusted difference (95% CI) | -0.23 (-0.55, 0.10) | -0.00 (-0.24, 0.23) | Reference | -0.03 (-0.33, 0.28) | -0.01 (-0.30, 0.28) |
|  | P | 0.18 | 1.00 |  | 0.86 | 0.95 |
| *Medical interview and professionalism (MP)* | | | |  |  |  |
|  | Mean±SD | 2.73±1.27 | 2.94±1.18 | 3.00±1.25 | 2.93±1.12 | 2.95±1.23 |
|  | Difference (95% CI) | -0.27 (-0.44, -0.10) | -0.06 (-0.18, 0.07) | Reference | -0.07 (-0.24, 0.09) | -0.07 (-0.22, 0.08) |
|  | P | 0.00 | 0.39 |  | 0.38 | 0.37 |
|  | Adjusted difference (95% CI) | -0.25 (-0.43, -0.07) | -0.04 (-0.17, 0.08) | Reference | -0.07 (-0.24, 0.10) | -0.07 (-0.23, 0.08) |
|  | P | 0.01 | 0.50 |  | 0.41 | 0.35 |
| *Disease Knowledge (DK)* | | |  |  |  |  |
|  | Mean±SD | 8.07±2.35 | 8.11±2.20 | 8.36±2.20 | 8.54±1.97 | 8.46±2.36 |
|  | Difference (95% CI) | -0.27 (-0.58, 0.04) | -0.26 (-0.49, -0.03) | Reference | 0.15 (-0.15, 0.46) | 0.08 (-0.20, 0.36) |
|  | P | 0.09 | 0.03 |  | 0.31 | 0.57 |
|  | Adjusted difference (95% CI) | -0.16 (-0.48, 0.16) | -0.22 (-0.45, 0.01) | Reference | 0.13 (-0.17, 0.43) | 0.01 (-0.27, 0.29) |
|  | P | 0.34 | 0.07 |  | 0.41 | 0.94 |

Note: Adjusted for gender, monthly Emergency Department (ED) duties, number of assigned inpatients, and self-study time. Non-responders for Emergency Department duties and inpatients were included in “unknown.” Non-responders for self-study time were excluded from the multivariable analysis. Abbreviations: C1–C5 = Category 1 to Category 5; SD = standard deviation; CI = confidence interval.

**Appendix S3**

**Supplemental Table 4**. Baseline characteristics of the university hospital residents

| Variable |  | University hospital residents |
| --- | --- | --- |
|  |  | N=840 |
| Male sex (%) |  | 545/ |
| ED duties per month (%) | |  |
| None |  | 11.7% |
| 1–2 |  | 27.4% |
| 3–5 |  | 53.2% |
| 6 or more |  | 4.9% |
| Unknown |  | 2.6% |
| Assigned inpatients (%) | |  |
| 0–4 |  | 21.5% |
| 5–9 |  | 51.5% |
| 10–14 |  | 18.0% |
| 15 or more |  | 5.8% |
| Unknown |  | 2.7% |
| Self-study time per day (%) | |  |
| None |  | 6.2% |
| 0–30 min |  | 42.4% |
| 31–60 min |  | 38.2% |
| 61–90 min |  | 11.5% |
| 91 min or more | | 1.5% |

Note: PGY = postgraduate year; ED = emergency department.

**Appendix S4**

**Supplemental Table 5**. Summary of the examination scores

|  | **GM-ITE score** |
| --- | --- |
|  | (mean ± SD) |
| **All residents** | 29.4±2.0 |
| **Hospital type** |  |
| University hospital | 27.6±5.3 |
| Community hospital | 29.7±5.3 |
| **Postgraduate year** |  |
| PGY-1 | 29.0±5.2 |
| PGY-2 | 29.8±5.6 |
| **Duty hours** |  |
| Category 1: <50 h | 28.1±5.6 |
| Category 2: 50–60 h | 29.2±5.3 |
| Category 3: 60–70 h | 29.7±5.3 |
| Category 4: 70–80 h | 29.8±5.2 |
| Category 5: >80 h | 30.0±5.7 |

Note: In this summary of the examination scores, we included not only community hospital residents, but also university hospital residents, who were excluded from this study’s analysis. GM-ITE = General Medicine In-training Examination; SD = standard deviation; PGY = postgraduate year.
